# RKIP在肺鳞癌组织中的表达及其意义

**DOI:** 10.3779/j.issn.1009-3419.2011.03.03

**Published:** 2011-03-20

**Authors:** 海源 徐, 馨 王, 文香 沈, 李强 王, 刚 沈, 敏斌 陈, 丽娜 周

**Affiliations:** 1 215300 昆山，江苏大学附属昆山医院，昆山市第一人民医院肿瘤科 Department of Medical Oncology, the First People's Hospital of Kunshan, Affiliated to Jiangsu University, Kunshan 215300, China; 2 361004 厦门，厦门大学附属中山医院肿瘤科 Department of Medical Oncology, Zhongshan Hospital, Affiliated to Xiamen University, Xiamen 361004, China

**Keywords:** 肺肿瘤, Raf激酶抑制蛋白, 转移, Lung neoplasms, Raf kinase inhibitor protein, Metastasis

## Abstract

**背景与目的:**

Raf激酶抑制蛋白（Raf kinase inhibitor protein, RKIP）属于磷脂酰乙醇胺结合蛋白家族的成员，可干扰Raf-1-MEK1/2-ERK1/2信号通路，抑制NF-κB和G蛋白偶联受体激酶等信号传导过程，且RKIP的表达减弱或丢失与多种肿瘤的发生发展以及侵润转移相关。本研究旨在探讨RKIP在肺鳞癌组织中的表达及其与肺鳞癌临床病理的相关性。

**方法:**

应用RT-PCR方法检测56例肺鳞癌组织及其癌旁正常组织中RKIP基因的表达，计算其阳性率；并用Western blot方法检测肺鳞癌组织及其癌旁正常组织中RKIP蛋白的表达变化。

**结果:**

RKIP mRNA在肺鳞癌组织中表达的阳性率明显低于癌旁正常组织（*P* < 0.05），在有淋巴结转移者中的表达阳性率明显低于无淋巴结转移者（*P* < 0.05），但与患者的性别、年龄、肿瘤的大小及分化程度无明显关系（*P*>0.05）。RKIP蛋白在肺鳞癌组织中表达明显低于癌旁正常组织（*P* < 0.05）。

**结论:**

RKIP的低表达可能与肺鳞癌的发生及侵袭转移有关。

## Abstract (en)

**Background and objective:**

Raf kinase inhibitory protein (RKIP) belongs to the phosphatidylethanolamine binding protein (PEBP) family. RKIP is an endogenous inhibitor of the Raf-1-MEK1/2-ERK1/2 signaling pathway, NFkappaB signaling pathway and G protein coupled receptors signaling pathway. The aim of this study is to explore the expression of RKIP in lung squamous cell carcinoma, and the relationship between RKIP expression and clinical pathology of lung squamous cell carcinoma.

**Methods:**

The expression of RKIP mRNA was detected in 56 lung squamous cell carcinoma tissues and adjacent cancer tissues by RT-PCR. The expression level of RKIP protein was detected by Western blot. The positive rates of RKIP expression in cancer tissues and adjacent cancer tissues were analyzed.

**Results:**

RKIP mRNA positive expression rate in cancer tissues was significantly lower than that in adjacent cancer tissues (*P* < 0.05). The positive rate of RKIP mRNA expression in the cancer tissues with lymph node metastasis was significantly lower than that without lymph node metastasis (*P* < 0.05). RKIP mRNA expression has no remarkable correlation with age, sex, tumor differentiation and size (*P*>0.05). RKIP protein expression in the cancer tissues was significantly lower than that in adjacent cancer tissues (*P* < 0.05).

**Conclusion:**

The deficiency of RKIP expression is positively correlated with carcinogenesis and invasion metastasis of lung squamous cell carcinoma.

Raf激酶抑制蛋白（Raf kinase inhibitor protein, RKIP）是近年来发现的一种新的肿瘤转移抑制因子，属于磷脂酰乙醇胺结合蛋白（phosphatidylethanolamine binding protein, PEBP）家族的成员，因发现PEBP能特异性地抑制Raf-1-MEK1/2-ERK1/2信号通路而将其命名为RKIP^[1]^。不同种属的PEBP家族成员都具有相似的结构域：即由一个大的β-折叠与连接在两侧的较小的β-折叠和两个C端α-螺旋组成。在该结构中存在一个高度保守的磷酸盐结合袋，对PEBP的功能非常重要^[1]^。人类*RKIP*基因定位于染色体12q24.23，其mRNA长1, 507 bp，由4个外显子转录产生。RKIP在哺乳动物的脑、心、肝、肺及睾丸组织中含量丰富，参与质膜生物合成、神经发育、精子发生、细胞凋亡等生理病理过程。近年来研究^[2, 3]^显示，RKIP不仅可以干扰Raf-1-MEK1/2-ERK1/2信号通路，而且还可以抑制NF-κB和G蛋白偶联受体激酶等信号传导过程，从而废除细胞的生存和抗凋亡特性。2003年Fu等^[4]^研究发现PKIP可以抑制肿瘤细胞转移的作用后，PKIP成为肿瘤领域的一个新的热点。最近研究资料^[4-8]^还显示RKIP的表达减弱或丢失与多种肿瘤的发生发展以及侵润转移相关，在前列腺癌、黑色素瘤、结直肠癌、肝癌、乳腺癌以及相关转移癌中的表达均低于其在正常对照组中的表达。但有关RKIP是否在肺鳞癌中表达以及与肺鳞癌发生发展关系的研究仍未见相关报道。我国是世界上肺癌患者最多的国家，且发病率和病死率一直呈上升趋势。近年来，尽管在肺癌治疗方面有了很大的进展，但肺癌生存率仍然不容乐观^[9]^。其主要原因是肺癌生物学特性十分复杂，恶性程度高，且80%肺癌为肺鳞癌，此类患者在确诊时已属晚期，大部分患者已发生转移，预后甚差。为此，我们必须寻找更加精确、有效的分子标志物，以期早期发现肿瘤。本研究通过RT-PCR和Western blot方法检测肺鳞癌组织及其癌旁正常组织中RKIP的表达差异，探讨RKIP与肺鳞癌的发生发展以及侵润转移的关系，以期为肺癌的预后判断以及靶向治疗等提供科学资料。

## 1 材料与方法

### 1.1 标本

收集江苏大学附属昆山医院和厦门大学附属中山医院2007年6月-2010年6月收治的肺鳞癌患者手术切除的新鲜标本56例，每例标本取材癌组织及其邻近的正常组织，立即置液氮中保存备用。其中男性32例，女性24例，年龄26岁-75岁，平均56岁，其中 < 60岁者36例，≥60岁者20例。术前未经任何治疗，病理学诊断56例肺鳞癌均为鳞癌。分化程度：高分化17例，中分化21例，低分化18例；有淋巴结转移39例，无淋巴结转移17例；肿瘤原发灶 < 3 cm者23例，≥3 cm者33例。

### 1.2 主要试剂

Trizol试剂盒（INVITROGEN公司），RTPCR试剂盒（GIBCOBRL公司），RIPA裂解液[1%NP-40, 1%Deoxycholate, 0.1%SDS, 500 mmol/L Tris, 150 mmol/L NaCl, 1 mmol/L PMSF, 19 Protease Inhibitor Cocktail (Roche, New Jersey, USA)]，TBST（10 mmol/L Tris-HCl, pH7.5, 150 mmol/L NaCl, 0.1%Tween-20），RKIP（NM_002567.2）上游引物：5'-AAGAATAGACCCACCAGCAT-3’，下游引物：5’-AACCAGCCAGACATAGCG-3’，预计扩增片段长度为234 bp；GAPDH（NG_007073.2）上游引物：5'-AATCCCATCACCATCTTCC-3'，下游引物：5'-CATCACGCCACAGTTTCC-3'，预计扩增片段长度为382 bp。所有引物序列均由上海英俊生物工程有限公司提供。RKIP一抗为兔抗人多克隆抗体（Invitroge公司，美国），二抗为HRP标记的山羊抗兔多克隆抗体（Abcam公司，英国），内参为单克隆小鼠抗人GAPDH抗体（Abcam公司，英国），二抗为HRP标记的山羊抗小鼠多克隆抗体（Abbiotec公司，美国），ECL发光液（Pierce公司，美国），BCA蛋白浓度测定试剂盒为国产碧云天产品。

### 1.3 采用RT-PCR检测RKIP mRNA的表达变化

按照试剂盒的操作说明提取癌组织和癌旁组织总RNA，取50 mg组织，加入1 mL Trizol试剂，匀浆，然后加入0.2 mL氯仿，振荡20 s，静置3 min。12, 000 rpm离心10 min，取上清；加入0.5 mL异丙醇，轻轻混匀，室温静置10 min。15, 000 rpm离心10 min，弃上清。加入1 mL的75%乙醇，轻轻洗涤沉淀，离心，弃上清；然后晾干，加入20 μL的DEPC水溶解（65 ℃促溶10 min-15 min）。然后进行RT反应。在20 μL的反应体积中加入2.0 μL总RNA，37 ℃孵育1 h，接着95 ℃灭活5 min，再立即冰浴，-20 ℃保存备用。进而设计引物，接着进行PCR反应，在25 μL PCR反应体系中加入cDNA 5 μL，扩增条件为：94 ℃预变性3 min，94 ℃变性45 s，57 ℃退火45 s，72 ℃延伸45 s；循环30次。最后72 ℃延伸10 min，经1.5%琼脂糖凝胶电泳鉴定PCR产物。

### 1.4 Western blot检测RKIP蛋白的表达变化

将液氮冻存的组织样品研磨，然后用RIPA裂解液进行匀浆，16, 000 g离心30 min，取上清，用BCA法测上清蛋白浓度，在每一个病例样品中取10 μg混合作为肺鳞癌组织蛋白样品，癌旁组织蛋白样品也是由每一个癌旁蛋白样品分别取10 μg混合而成。制备12%SDS-PAGE，每泳道加总蛋白量40 μg，进行电泳，经半干转印至PVDF膜（Amresco公司，美国），在含有5%脱脂奶粉的TBST溶液中常温封闭1 h，然后分别加入RKIP兔抗人多克隆抗体（1:1, 000稀释）及GAPDH小鼠抗人单克隆抗体（1:1, 000稀释），4 ℃孵育过夜，然后加入HRP标记的山羊抗兔IgG（1:2, 000稀释）和HRP标记的山羊抗小鼠多克隆抗体（1:2, 000稀释），37 ℃孵育1 h，TBST洗涤，ECL化学发光试剂自显影。RKIP相对含量用RKIP/GAPDH灰度比值表示，灰度采用QuantityOne软件（Bio-Rad公司，美国）分析。

### 1.5 统计学分析

采用Stata 7.0统计软件进行*χ*^2^检验或t检验，以*P* < 0.05为有统计学差异。

## 2 结果

### 2.1 肺鳞癌组织中RKIP mRNA表达

RT-PCR产物经1.5%琼脂糖凝胶电泳检测见一特异扩增条带，其分子量大小与预期结果相符（图 1）。RKIP mRNA在肺鳞癌组织中的表达情况见表 1。即RKIP基因在肺鳞癌组织中表达阳性率明显低于正常组织（*P* < 0.05），在有淋巴结转移者中的表达阳性率明显低于无淋巴结转移者（*P* < 0.05），但与患者性别、年龄及肿瘤的大小及分化程度无关（*P*>0.05）。

**1:**
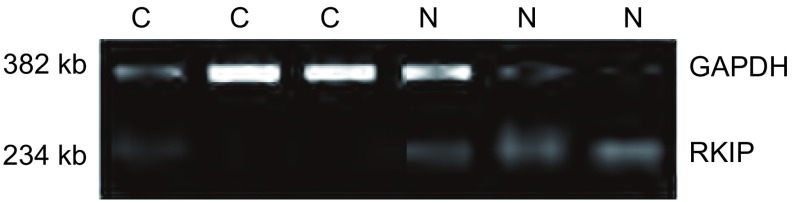
肺鳞癌组织及癌旁组织中RKIP mRNA的表达。N：癌旁组织；C：肺鳞癌组织 The expression of RKIP mRNA in lung squamous cell carcinoma tissues and adjacent cancer tissues. N: adjacent normal tissues; C: squamous cell carcinoma tissues

**1:** 肺鳞癌组织中RKIP mRNA表达与临床病理因素的关系 The relation between expression of RKIP mRNA in lung squamous cell carcinoma tissues and clinicopathologic factors

Pathologic parameter	*n*	RKIP mRNA positive rate [*n* (%)]	*χ*^2^	*P*
Tissue			8.851	0.003
Adjacement cancer tissue	56	44 (78.57)		
Squamous cell carcinoma tissue	56	29 (51.79)		
Sex			0.054	0.817
Male	32	17 (53.13)		
Female	24	12 (50.00)		
Age (year)			2.176	0.140
< 60	36	16 (44.44)		
≥60	20	13 (65.00)		
Size of primary carcinoma (cm)			1.290	0.256
< 3	23	14 (60.87)		
≥3	33	15 (45.45)		
Degree of differentiation			0.258	0.879
Well differentiated	17	8 (47.06)		
Moderately differentiated	21	11 (52.38)		
Poorly differentiated	18	10 (55.56)		
Lymph nodes metastasis			12.989	< 0.001
Negative	17	15 (88.24)		
Positive	39	14 (35.90)		

### 2.2 肺鳞癌组织及癌旁组织中RKIP蛋白的表达

RKIP蛋白在肺鳞癌组织中的表达量明显低于癌旁正常组织（*P*=0.037, 1）（图 2）。

**2:**
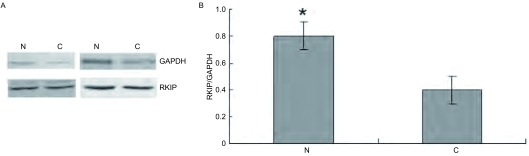
肺鳞癌组织及癌旁组织中RKIP蛋白的表达。A：Western blot结果；B：RKIP蛋白在肺鳞癌组织及癌旁组织中的相对表达变化。^*^：癌旁组织与肺鳞癌组织相比，*P* < 0.05 The expression of RKIP in lung squamous cell carcinoma tissues and adjacent cancer tissues. A: Results of Western blot; B: The relative expression changes of RKIP in squamous cell carcinoma tissues and adjacent cancer tissues. ^*^: adjacent cancer tissues vs squamous cell carcinoma tissues, *P* < 0.05

## 3 讨论

肺癌为当前世界各地最常见的恶性肿瘤之一，是一种严重威胁人类健康和生命的疾病。近半个世纪以来，世界各国肺癌的发病率和病死率都有明显的增高。全球每年新增肺癌病例将达120万。我国是世界上肺癌患者最多的国家，且发病率和病死率一直呈上升趋势。近30年来，尽管在肺癌治疗方面有了很大的进展，但肺癌生存率仍然不容乐观^[9]^。其主要原因是肺癌生物学特性十分复杂，恶性程度高，且80%肺癌为肺鳞癌，此类患者在确诊时已属晚期，大部分患者已发生转移，预后甚差。为此，我们必须寻找更加精确、有效的分子标志物，以期早期发现肿瘤。

RKIP是近年来发现的一种新的肿瘤转移抑制因子，广泛表达于哺乳动物的脑、心、肝、肺及睾丸等组织，参与质膜生物合成、神经发育、精子发生、细胞凋亡等生理病理过程。近年来研究^[2, 3]^显示，RKIP不仅可以干扰Raf-1-MEK1/2-ERK1/2信号通路，而且还可以抑制NF-κB和G蛋白偶联受体激酶等信号传导过程。自2003年Fu等^[4]^研究发现PKIP可以抑制肿瘤细胞转移的作用后，PKIP成为肿瘤领域的一个新的热点。Lee等^[5, 10]^研究发现RKIP在肝癌中的表达明显低于邻近的正常肝组织，增加RKIP的表达可以减少肝癌细胞的增殖和迁移。RKIP在甲状腺癌中的表达低于正常的甲状腺组织，外源性的RKIP可以抑制甲状腺癌细胞的生长^[11]^。还有研究^[8, 12]^发现PKIP在乳腺癌的原发病灶中高表达，其表达与乳腺癌的类型、分化程度、大小、ER的表达无关，但在转移淋巴结中低表达或无表达；RKIP在正常黑色素细胞中表达量最高，黑色素瘤次之，黑色素瘤转移灶最低或缺失；过表达RKIP可以抑制高转移性黑色素瘤细胞的转移能力^[6]^。本研究通过检测肺鳞癌组织及其癌旁正常组织中*RKIP*基因的表达发现：RKIP mRNA在肺鳞癌组织中表达的阳性率明显低于癌旁正常组织（*P* < 0.05），在有淋巴结转移者中的表达阳性率明显低于无淋巴结转移者（*P* < 0.05），但与患者的性别、年龄、肿瘤的大小及分化程度无关（*P*>0.05）；RKIP蛋白在肺鳞癌组织中表达也明显低于癌旁正常组织（*P* < 0.05）。可见RKIP在肺鳞癌组织中表达结果与其它肿瘤相似。

RKIP可破坏Raf-1和MEK-1形成的复合物，使Raf-1脱离MEK-1。RKIP可以分别与Raf-1和MEK-1结合，当与其中任意一个结合时就足以抑制下游信号^[13]^，而在本研究中发现RKIP在肺鳞癌组织中明显表达减弱或缺失，这就大大削减了RKIP对Raf-1-MEK1/2-ERK1/2信号通路的抑制作用，从而可以促进细胞的生长和发育，这可能与肺鳞癌的发生相关。另外，RKIP还可以通过与NF-κB激活途径的4个激酶NIK（NF-κB inducing kinase）、TAKI（TGF-β activated kinase 1）、IKKα（IκB kinase α）和IKKβ（IκB kinaseβ）相互作用，从而抑制NF-κB激活，减弱细胞的抗凋亡特性^[1]^。RKIP在肺鳞癌组织中明显表达减弱或缺失，可以促进NF-κB通路的激活，增加细胞的抗凋亡性能，这可能与肺鳞癌的发生发展相关。Beshir等^[2]^研究发现，RKIP可以控制基质金属蛋白酶1/2（MMP-1/2）的表达，从而发挥其促进肿瘤细胞侵润转移的功能；沉默RKIP后肿瘤细胞的侵润转移能力增强，MMP-1/2表达水平增高；过表达RKIP后肿瘤细胞的侵润转移能力减弱，MMP-1/2表达水平降低。本研究发现RKIP在有淋巴结转移肿瘤组织中的表达阳性率明显低于无淋巴结转移者（*P* < 0.05）。RKIP在肺鳞癌组织中表达减弱或缺失，可能增加了MMP-1/2的表达，从而促进了肺鳞癌细胞的侵润转移，具体机制尚待进一步验证。

综上，RKIP的表达减弱或缺失在肺鳞癌的发生发展以及侵润转移过程中可能起着重要的作用。这或许可以为肺鳞癌的治疗提供一个潜在的靶标。
